# Clinical assessment is a neglected component of outbreak preparedness: evidence from refugee camps in Greece

**DOI:** 10.1186/s12916-018-1015-9

**Published:** 2018-03-19

**Authors:** Amanda M. Rojek, Kassiani Gkolfinopoulou, Apostolos Veizis, Angeliki Lambrou, Lyndsey Castle, Theano Georgakopoulou, Karl Blanchet, Takis Panagiotopoulos, Peter W. Horby, The Epidemic Diseases Research Group field team, Angeliki Angeletopoulou, Persephoni Dimitsaki, Angeliki Karatza, Athina Kourouni, Maria Moschovou, Evangeli Petroudi, Artemis Stoli

**Affiliations:** 1 Epidemic Diseases Research Group, University of Oxford, Wellcome Trust Centre for Human Genetics, Oxford, UK; 2 Hellenic Centre for Disease Prevention and Control (KEELPNO), Agrafon 3-5, Athens, Greece; 3 Médecins Sans Frontières, 15 Xenias St, Athens, Greece; 4 Health in Humanitarian Crises Centre, London School of Hygiene and Tropical Medicine, Keppel St, London, WC1E 7HT UK; 5 National School of Public Health, Leof. Alexandras, 196 Athens, Greece

**Keywords:** Refugee, Outbreak, Infectious disease, Syndromic surveillance, Epidemic

## Abstract

**Background:**

Refugees may have an increased vulnerability to infectious diseases, and the consequences of an outbreak are more severe in a refugee camp. When an outbreak is suspected, access to clinical information is critical for investigators to verify that an outbreak is occurring, to determine the cause and to select interventions to control it. Experience from previous outbreaks suggests that the accuracy and completeness of this information is poor. This study is the first to assess the adequacy of clinical characterisation of acute medical illnesses in refugee camps. The objective is to direct improvements in outbreak identification and management in this vulnerable setting.

**Methods:**

We collected prospective data in 13 refugee camps in Greece. We passively observed consultations where patients presented with syndromes that might warrant inclusion into an existing syndromic surveillance system and then undertook a structured assessment of routine clinical data collection to examine the extent to which key clinical parameters required for an outbreak response were ascertained and then documented.

**Results:**

A total of 528 patient consultations were included. The most common presenting condition was an acute respiratory illness. Clinicians often made a comprehensive clinical assessment, especially for common syndromes of respiratory and gastrointestinal conditions, but documented their findings less frequently. For fewer than 5% of patients were a full set of vital signs ascertained and so the severity of patient illnesses was largely unknown. In only 11% of consultations was it verified that a patient who met the case criteria for syndromic surveillance reporting based on an independent assessment was reported into the system.

**Discussion:**

Opportunities exist to strengthen clinical data capture and recording in refugee camps, which will produce a better calibrated and directed public health response.

**Conclusion:**

Information of significant utility for outbreak response is collected at the clinical interface and we recommend improving how this information is recorded and linked into surveillance systems.

**Electronic supplementary material:**

The online version of this article (10.1186/s12916-018-1015-9) contains supplementary material, which is available to authorized users.

## Background

Refugees arriving in Europe may be especially vulnerable to infectious diseases for reasons including poor rates of vaccination, poor immunity to endemic diseases in regions of movement, malnutrition, lack of access to safe drinking water, or living in over-crowded or unhygienic conditions [1–3]. These risk factors can occur in the country of origin, during transit or during early settlement [2, 3]. While definitive population-wide estimates are not available for refugees arriving within the European Union (EU) [4–9], respiratory tract infections were the most common medical problem diagnosed (prevalence of 23%) amongst 6899 refugees arriving at the Greek border with Turkey in one assessment [10]. Médecins Sans Frontières clinics at refugee points of entry into Greece and Serbia diagnosed respiratory tract infections in 41% of patients (*n* = 33,331) accessing care [11].

Given the present scale of global migration, these infections can constitute a significant treatment burden. In Turkey in 2015, there were an excess of 330,000 cases of respiratory tract infections, and 50,000 diarrhoea cases amongst the 2.7 million refugees hosted from Syria [1]. The consequences of an outbreak in a refugee camp are exacerbated. For example, novel influenza in this setting is predicted to cause complication rates double that of the general population [12]. It is important to note that this risk in refugee populations does not imply a risk of ongoing transmission to the hosting community [3].

Despite this evident vulnerability, few works have assessed the capability to identify disease outbreaks rapidly and correctly in this setting. When the World Health Organization (WHO) developed their outbreak surveillance, investigation and response (OSIR) guidelines for humanitarian settings, they relied on expert advice in light of the paucity of quantitative data [13]. One important step in comprehensive OSIR systems is alert verification. When an alert or signal is produced by the system, epidemiologists (or other personnel) must decide whether it represents an event of public health importance. They may then plan further investigation and determine an appropriate response. This step is heavily reliant on accessing information regarding the clinical characterisation of cases, including the spectrum and severity of patient symptoms, and whether the population groups are those most frequently infected or experience the most severe disease. Initial information reporting the clinical characteristics of cases may be a very important information source for alert verification in settings where people are on the move and there is little opportunity for additional contact with them.

However, there is some evidence to suggest that these assessments may be especially difficult to achieve in a refugee camp. For example, an investigation of a shigellosis outbreak in a refugee camp in Greece may have underestimated the outbreak size due to difficulties in language, under-diagnosis of cases with mild symptoms, or denial of symptoms from patients unwilling to risk a delay to departure from the refugee camp [14].

The purpose of this work is to provide quantitative measures of what information may be available to outbreak response teams verifying and investigating a cluster of cases in a refugee camp. The findings of this work will provide an evidence-based framework to direct improvements in the alert verification and outbreak investigation components of OSIR systems. Importantly, this work directs improvement for refugee settings in advance of an outbreak.

## Methods

Prospective observational data were collected in 13 refugee camps in Greece from 3 July to 28 July 2017. The selection of camps was a convenience sample, with a preference for camps operating at the points of arrival of refugees into the country.

Our research team passively observed clinical consultations that were conducted as part of routine care in the refugee camp, and they collected data on the clinical information captured in that setting. Because the purpose of the project was to evaluate normal practice, the research team did not seek additional information from patients, or request additional history taking or examination from clinicians, or provide any feedback on the care provided.

Data were recorded for consultations where a refugee presented for the first time with a medical illness of recent onset (defined as within 1 month) that was not due to trauma or a known toxin. There were no exclusions based on patient age, gender, nationality or legal status.

The framework for assessment was based on categories of clinical information required for alert verification and outbreak investigation. These were an assessment of infectious disease exposures [we recorded three types of exposure: recent overseas arrival within 1 month, close contact (household or nursing) with an unwell contact and known or possible zoonotic exposure], indicators of infectious disease vulnerability (we recorded two common vulnerable groups: pregnant women and those with a co-morbid illness), the spectrum of clinical signs and symptoms observed (assessed according to those features included in the case definition for syndromes under syndromic surveillance) and the severity of presentations.

The severity of patient illness was assessed using two widely used standardised early warning scores: the United Kingdom’s National Early Warning Score (NEWS) [15] and the paediatric equivalent, the Children’s Observations and Severity Tool (COAST) [16, 17]. These scoring systems allocate points to physiologically abnormal vital signs to produce an aggregate score that is used to triage patients according to severity of presentation (irrespective of the underlying pathology). This score was modified to exclude scoring based on supplemental oxygen provision, as this is not routinely available in refugee camps. A description of the case definitions in the syndromic surveillance system used in refugee camps in Greece is provided in Additional file 1: Appendix 1 [18].

There were three components of data collection: (a) whether the variable was assessed or otherwise ascertained during the clinical consultation (including negative or normal findings), (b) whether the result (including negative or normal findings) were recorded in a written or electronic clinical record and (c) the diagnosis or result, if known.

Factors that may have contributed to the quality of information obtained in the consultation were recorded. These were the workload of the clinic, the type of health-care worker providing care and any language difficulties [19].

Observers were all registered nurses. All observers received one day of protocol-specific training from the principal investigator (AR). The accuracy of data collected by the observer was tested using standardised videotaped consultations. The mean accuracy of observer reporting was 95.8% (standard deviation 3.5%). To maintain accuracy further, observers were encouraged to select an unsure option if there was uncertainty in an assessment.

For this study, refugees, asylum seekers, migrants in an irregular situation and persons of unknown status are referred to using the collective term ‘refugee(s)’.

### Statistical analysis

Descriptive statistics are presented as frequencies for categorical variables, means and standard deviations for normally distributed data, and medians with a range for other continuous variables. The statistical software STATA (version MP 15) was used for statistical analysis.

### Ethics

The study protocol was granted an exemption by the University of Oxford ethics committee as it constitutes a clinical audit.

## Results

**Fig. 1:**
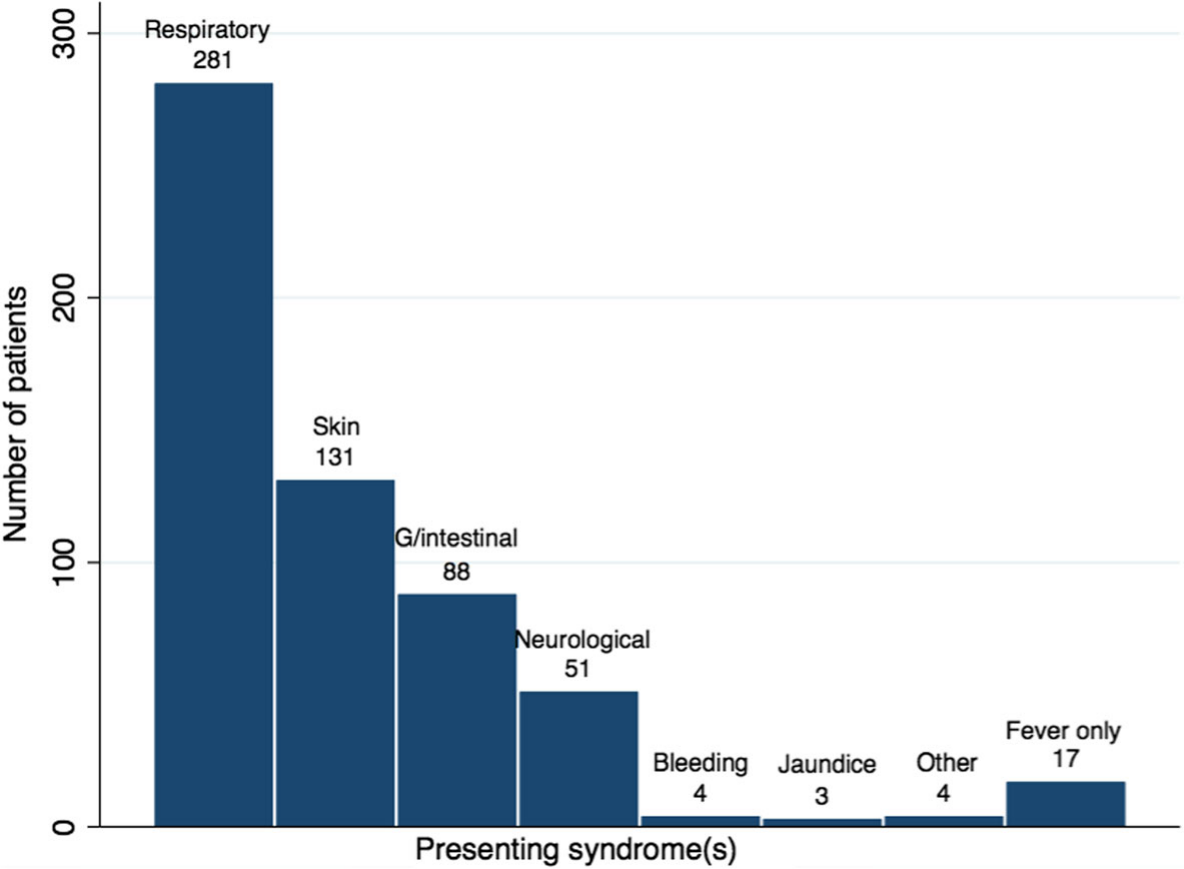
Number of refugee patients presenting with different syndromes. ‘Other’ refers to other syndromes not included in syndromic surveillance (such as urinary tract symptoms). G/intestinal gastrointestinal

A total of 528 patient consultations were included in the study. Of these, 306 patients were male. The median age of patients was 19 years (range 1 month to 70 years) (Additional file 2: Appendix 2). The most frequent reason for presentation was an acute respiratory tract illness, followed by a skin condition (Fig. 1).

### Assessment of exposure to infectious disease

**Fig. 2:**
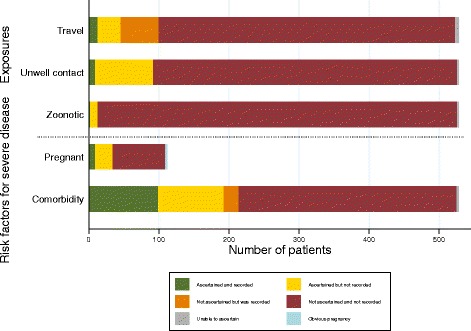
Assessment of possible exposure and vulnerability to infectious diseases. Exposure history includes recent travel (defined as international arrival within 1 month), unwell contacts (household contact or provided nursing care) and zoonotic exposure. Vulnerability includes pregnancy (women aged 12–50 years only) and any other medical condition

Figure 2 shows the extent to which important exposure to infectious disease (due to recent international travel, close contact with unwell contacts or zoonotic exposure) was ascertained and then recorded. There was no assessment of these risk factors in most consultations [97% (*n* = 513) for known or suspected zoonotic exposure, 82% (*n* = 434) for close contact with unwell contacts and 80% (*n* = 423) for international travel within 1 month]. However, when these risk factors were assessed and the finding was known, patients had zoonotic exposure in 38% of cases (*n* = 5), had recently (within 1 month) arrived in Greece in 32% of cases (*n* = 32) and had close contact with unwell persons in 58% of consultations (*n* = 54).

### Assessment of vulnerability to infectious disease

Figure 2 also displays the extent to which common risk factors for susceptibility or increased severity to infectious disease were assessed. Two broadly applicable risk factors were selected: pregnancy and co-morbid disease. There was no assessment of pregnancy status in at least 66% of consultation with women of child-bearing age (*n* = 75), and co-morbid conditions were not inquired about in at least 58% of all consultation (*n* = 311). When assessed and the finding was known, women of child-bearing age were pregnant in 21% of consultation (*n* = 7). When assessed, a patient had one or more co-morbidities in 28% of consultation (*n* = 60).

### Clinical characterisation

**Fig. 3:**
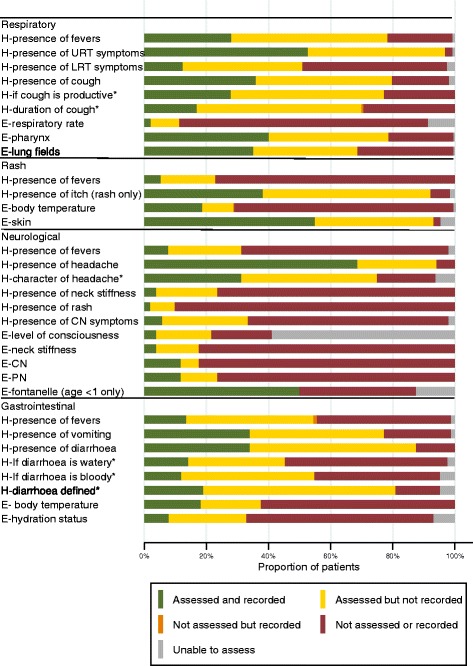
Assessment of clinical characterisation of presenting syndromes (limited to syndromes with >10 patients presenting). The clinical features assessed for each syndrome are based on case criteria for conditions under syndromic surveillance (either the platform used in refugee camps in Greece or that suggested by WHO for use in humanitarian settings). * means that this assessment only occurred when the primary symptom was present. E assessment by physical examination, CN cranial nerves, H symptoms assessed by history taking, PN peripheral nervous system, LRT lower respiratory tract, URT upper respiratory tract

Figure 3 shows the extent to which features of the most common presenting syndromes were ascertained and recorded (irrespective of the findings). Overall, clinicians did assess many of the key characteristics of syndromes, particularly for the common presentations of gastrointestinal conditions and respiratory conditions. However, in all but one variable (assessment for bulging fontanelles in infants presenting with a neurological syndrome), clinicians did not document all their clinical findings as frequently as assessment occurred.

Table 1 compares whether patients were observed to have met (or not met) the case definition criteria for notification in the syndromic surveillance system based on what was observed during consultations, with their actual inclusion or non-inclusion in the notification system. In 11% of cases (*n* = 59), there was agreement between the observer assessment and the actual reporting. In 23% of cases (*n* = 119), there was disagreement, meaning that the patient met all the criteria in the case definition (based on information ascertained or recorded during the observed consultation) but this was not reported, or was reported under a different syndrome. In the remaining 66% of cases (*n* = 350), there was inadequate information to make a comparison. This classification included cases where the patient did not meet the case definition based on observation, but the patient was still reported on the syndromic surveillance form. This was done, rather than considering these cases as a known disagreement, because the notification form always includes provision for a clinician to report a case due to clinical suspicion, even if the syndrome definition is not met. In 32% of cases (*n* = 169), observers were unable to link the consultation with reporting—either it was unknown whether the patient was reported, or it was unknown what syndrome they were reported with.

**Table 1:**
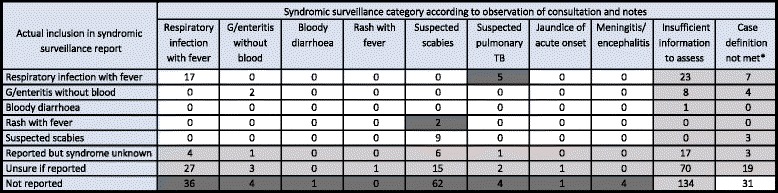
Assessment of clinical characterisation of syndromes

Comparison is made between the reporting of the patient in the syndromic surveillance system (rows) with whether that patient is believed to have met the case definition based on the information collected during the consultation (columns). White boxes indicate agreement between reporting status and assessment based on the case definition. Light grey boxes indicate a potential for discrepancy between reported status and case definition assessment and dark grey boxes indicate that the patient was not reported despite meeting the case definition. ‘Insufficient information to assess’ indicates that there was insufficient assessment to include or exclude a patient based on the case definition. *Potential discrepancy as inclusion may have been based on clinical suspicion

### Severity assessment

In very few patients (fewer than 4% of children and 1% of adults) were a full set of vital signs available to the observer (Table 2). Therefore, the extent to which the severity scores presented (for patients with one or more vital signs taken) reflect severity across the entire population is not known.

**Table 2:**
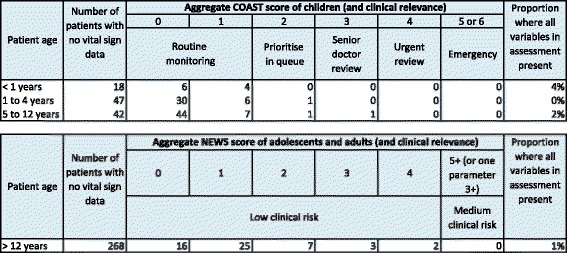
Assessment of severity of patient presentations

Two scoring systems based on vital signs are used: the National Early Warning Scoring system, which is used in adult patients and the Children’s Observations and Severity Tool for children, as described in ‘Methods’. Data are shown only for patients where one or more vital signs were recorded

### Factors that may have influenced consultation quality

The median time per consultation was 10 min (range 1–45 min). Our observers could not reliably document how busy or crowded the clinic was. Of consultations, 99% (*n* = 521) were undertaken by doctors and 1% (*n* = 7) by registered nurses. A professional interpreter or cultural liaison was used for 91% of consultations (*n* = 478), and a family member interpreted for 2% of consultations (*n* = 9). No interpreter was available, but language difficulties were experienced in 2% of consultations (*n* = 12). The clinician spoke the same language(s) as the patient in 6% of consultations (*n* = 29). Observers were asked to list other factors they believed may have affected the consultation and answers included multiple patient consultations occurring at the same time (1%, *n* = 5), other communication problems (1%, *n* = 5), a distressed patient or acute concerns about the patient’s mental health (<1%, *n* = 3), too many people (six or more) being present in the room (<1%, *n* = 2), the status of the patient as an unaccompanied minor (<1%, *n* = 1), constant interruptions (<1%, *n* = 1) and a patient being unwilling to have a physical examination (<1%, *n* = 1).

## Discussion

Here we report data on clinical information likely to be available for alert verification and outbreak investigation in a humanitarian setting.

We found that when acute medical syndromes were encountered in refugee camps, clinical characterisation of the predominant syndrome was, in general, performed well. For example, as shown in Fig. 3, 81% of the times when patients had diarrhoea (*n* = 34), it was defined in the consultation (as three or more loose stools per day), and the type of cough (productive or non-productive) was assessed in 77% of patients presenting with this symptom (*n* = 119). Despite this, the occasions when clinical assessment was not comprehensive led to difficulties in ascertaining if a patient was presenting with a syndrome under surveillance (as shown in Table 1).

With very few exceptions, case records did not contain all the information assessed during the consultation. This is an important opportunity missed, because clinicians are spontaneously ascertaining information that would be valuable for an outbreak team. Our concern is that in refugee camps, alternative means of investigating a case may not be readily available. Interviewing a patient or their family is often used in other situations, but this may not be accurate in a refugee camp as refugees may not wish to disclose symptoms [14]. Furthermore, the extent to which refugees who are migrating through Europe can be traced so that information can be verified is unknown. In addition, the high turnover of clinicians in some camps means these individuals may not be accessible to outbreak investigators either. Tools to facilitate the rapid recording of information should be urgently developed.

We report low ascertainment of exposure to infectious diseases. This is despite the differential diagnosis for illnesses in refugees in Europe varying by their country of origin, transit route and living conditions [3]. For well-characterised diseases, identification of the source of the outbreak directs control. For an emerging disease, it is integral for hypothesis generation. Likewise, we found incomplete assessment of whether refugees belonged to a population vulnerable to infectious diseases. These findings agree with reports that statutory surveillance systems for refugee populations in Europe cannot stratify patient populations during outbreaks [20]. This impacts the prioritisation of resources and care during an outbreak.

Our assessment of the severity of patient illness found that full set of vital signs were taken for fewer than 5% of patients in any age group, which limits the external objective assessment of patient severity. This finding is not unexpected. The lack of consistent and understandable measures of patient severity during the H1N1pdm09 pandemic was one of the most notable failures of the outbreak, which resulted in an overestimation of disease severity [21]. While there are other means of assessing patient severity, we used a widely used and validated early warning system.

We do not suggest that patients assessed to meet the case criteria for a notifiable disease, but who were not reported, were misdiagnosed. There are various valid reasons why this may occur (such as there being a clear alternative explanation for their symptoms or signs). However, universal adoption and strict adherence to case definitions is encouraged by public health authorities, which expect false positive notifications as this is a preferable outcome to under-reporting [13]. Another plausible explanation for under-reporting may be poor familiarisation with the case definitions or low motivation to report systematically. Therefore, far from identifying errors in clinician practice, our findings of discrepancies between assessor reporting and clinician reporting are instead broadly illustrative of common difficulties in interpretation and use of syndromic surveillance between different stakeholders. The extent to which uncertainty in the diagnosis and inclusion of an individual patient affects the syndromic surveillance system cannot be ascertained from our findings. It is expected that the function of the system is somewhat resilient to both false positive and false negative reports, especially if these occur at a constant rate.

Our understanding of factors that impede comprehensive patient evaluation is limited. Unfortunately, we could not reliably measure how busy clinics were. For the majority of consultations, language did not appear to be an issue, but there were reports of other communication difficulties during consultations. Frequent interruptions and multiple simultaneous consultations are likely to impair consultation quality, but do reflect the reality of the working environment.

### Limitations

The scope of this work was limited to patient presentations in official refugee camps in Greece. The representativeness of these findings for refugees treated in other health-care settings or other countries is unknown. Furthermore, we treat refugees as a homogeneous presenting group, although the risk of infectious diseases could differ within this population. During the period of our study, there were no outbreaks of immediately notifiable syndromes (such as acute paralysis) and so clinical assessment for these presentations could not be assessed. We used a broad classification to identify patients with presenting syndromes that may include an infectious disease. It is possible, for some of these patients, that the presenting syndrome clearly posed no possibility of being an infectious disease. However, a sub-group analysis was not possible. While we attempted to perform this based on febrile status, there was an insufficient proportion of cases where fever was ascertained, and some syndromes do not require fever in their case definition. We could not assess for malaria, diphtheria or sepsis, which are reportable syndromes in Greek refugee camps, as ascertainment of some elements of the case criteria could not be achieved by passive observation (e.g. the presence of a pharyngeal pseudo-membrane for diphtheria) or they relied on laboratory diagnosis. In retrospect, presenting neurological syndromes should have been divided into headache and other nervous system presentations (such as acute paralysis or weakness), as the types of clinical assessment required for diagnosis differ significantly between these presentations. We were interested in clinician assessment of patient vaccination status, but could not do so due to the wide variability in how this was assessed.

### Future directions

Opportunities exist to improve the clinical information available for OSIR in refugee camps. A focused examination of the barriers that clinicians experience in generating better evidence is warranted. We have now convened an expert working group with the objective of adopting or producing tools and methods that facilitate the acquisition and recording of clinical information that meet the needs of both clinicians and public health responders.

## Conclusions

Our findings suggest that the performance of OSIR in humanitarian settings can be enhanced if work is undertaken to ensure that comprehensive and accurate clinical information is available for verifying and investigating alerts. Specifically, this requires improved documentation of information that is obtained during patient consultations and improving clinicians’ understanding and utilisation of surveillance tools (such as case definitions for syndromes under surveillance).

## Additional files

Additional file 1: Surveillance reporting form for refugees at point of care in Greece. (PDF 638 kb)

Additional file 2: Demographic data for included patients. (PDF 12 kb)

## Abbreviations

COAST: Children’s Observations and Severity Tool
EU: European Union
LRT: Lower respiratory tract
NEWS: National Early Warning Score
OSIR: Outbreak Surveillance, Investigation, and Response
URT: Upper respiratory tract
WHO: World Health Organization
