# Supplementary material for: Clinical assessment is a neglected component of outbreak preparedness: evidence from refugee camps in Greece
Source: BMC Med. 2018 Mar 19;16:43. doi: 10.1186/s12916-018-1015-9 (PMC5858141; doi:10.1186/s12916-018-1015-9)
Supplement: Supplementary file 1 — Surveillance reporting form for refugees at point of care in Greece. (PDF 638 kb) [file 12916_2018_1015_MOESM1_ESM.pdf]

MINISTRY OF HEALTH
Centre for Disease Control and Prevention (KEELPNO)
Department of Surveillance and Intervention

Contact information:
Tel: **210 5212 054**, Fax: **210 8818 868**
E-mail: epid@keelpno.gr

(Epidemiologic surveillance according to Law 3370/2005)

# REPORTING FORM – SURVEILLANCE IN POINTS OF CARE FOR REFUGEES/MIGRANTS

(Separate Reporting Forms are required for every clinic per 24hr-period, hours 00:01 to 24:00. To be submitted by 09:00 on the next day.)

Page:

| | | | |
|---|---|---|---|
| **Site-Clinic:** | | **Date:** | |
| **Organization(s):** | | **Clinic hours (from-to):** | |
| **Name of health professional:** | | | |

> Please **CALL IMMEDIATELY** KEELPNO (210 5212 054) in case of clinical suspicion of "immediately notifiable diseases" (see Instructions) or in case of cluster of cases with unusual or severe manifestations.

## SYNDROMES OR CONDITIONS UNDER SURVEILLANCE ([1] to [14]):

| Table (A) | 0–4 yrs | No. | 5–17 yrs | No. | 18+ yrs | No. | Total |
|---|---|---|---|---|---|---|---|
| [1] Respiratory infection WITH fever | | | | | | | |
| [2] Gastroenteritis without blood in the stool | | | | | | | |
| [3] Bloody diarrhoea | | | | | | | |
| [4] Rash WITH fever | | | | | | | |
| [5] Suspected scabies | | | | | | | |

| Table (B) | No. | | No. |
|---|---|---|---|
| [6] Suspected pulmonary tuberculosis | | [10] Paralytic manifestations of acute onset | |
| [7] Malaria (with POSITIVE rapid test) | | [11] Meningitis and/or encephalitis | |
| [8] Suspected diphtheria, respiratory or cutaneous | | [12] Haemorrhagic manifestations WITH fever | |
| | | [13] Sepsis or shock (septic, of unknown aetiology) | |
| [9] Jaundice of acute onset | | [14] Death of unknown aetiology | |

NOTE: Table (A): the cell corresponding to syndrome and age group can be used to mark cases (e.g. 𝍸I), in order to facilitate counting.
Tables (A) and (B): In cell "No." the total number of cases (e.g. 6) is included. **If there are no cases write "0" in the cell (zero reporting).**

> Please **fill-in Table C**:

- For cases **included in Table (B)**, and
- For cases with clinical suspicion of **measles**, **rubella**, **mumps (parotitis)**, **chickenpox (varicella)**.

## Table (C)

| # | Patient Surname-Name, Dwelling, Contact telephone no. | Gender | Age | Country of origin | # of Syndrome | Suspected disease [if relevant] | Transfer hospital [if relevant] | Examining physician in site & Contact tel. |
|---|---|---|---|---|---|---|---|---|
| | | M F | | | | | | |
| | | M F | | | | | | |
| | | M F | | | | | | |
| | | M F | | | | | | |

*If needed, continue in table (C) of another Reporting Form (mark Page at the top right of Forms)*

| Table (D) | 0–4 yrs | 5–17 yrs | 18+ yrs | Total |
|---|---|---|---|---|
| TOTAL NUMBER OF CLINIC VISITS (FOR ALL CAUSES) | | | | |
| TOTAL NUMBER OF PERSONS IN THE CENTRE (ON THE SAME DAY) | | | | |

**> Information on Form was sent (check box):** ☐ Sent by: Date:

*FORM UPDATE: 16 May 2016*

MINISTRY OF HEALTH
Centre for Disease Control and Prevention (KEELPNO)
Department of Surveillance and Intervention

Contact information:
Tel: **210 5212 054**, Fax: **210 8818 868**
E-mail: epid@keelpno.gr

(Epidemiologic surveillance according to Law 3370/2005)

# INSTRUCTIONS FOR COMPLETING THE SURVEILLANCE FORM

***Call immediately KEELPNO in case of clinical suspicion of "immediately notifiable diseases":***
(1) Anthrax, (2) Botulism, (3) Cholera, (4) Diphtheria, (5) Encephalitis/arboviral,
(6) Haemorrhagic fever, (7) Plague, (8) Rabies, (9) Toularaemia.

## Initial information

| | |
|---|---|
| Site-Clinic | Include site (Centre) hosting refugees/migrants and name or other identification of clinic (if there are more than one clinics in the site). |
| Organization(s) | Include the Organization which runs the clinic (or the Organizations, if more than one) |
| Name of health professional | Each physician on duty at the clinic writes his/her name (more than one names can be included), so that –if need arises– communication with him/her for clarifications on the clinical presentation of syndromes/conditions is possible. |
| Clinic hours (from-to) | Total clinic hours in the 24hr period are included, irrespective of number of physicians involved. |

## Definition of syndromes or conditions under surveillance

**[1] Acute respiratory infection WITH fever**
Either (A) or (B), or both
**(A)** - Fever (≥38.0°C directly measured or reported by the patient)
AND at least one of the following:
- Manifestations of upper respiratory tract infection: e.g. runny nose, nasal congestion, sore throat, cough, ear drum inflammation
- Manifestations of lower respiratory tract infection: e.g. cough, breathing difficulty, chest rales, increased respiratory rate

**(B)** Clinical suspicion of viral nasopharyngitis, influenza (ILI), pharyngitis/tonsillitis, otitis media, sinusitis, tracheitis, bronchitis, pneumonia, bronchopneumonia, bronchiolitis.

**[2] Gastroenteritis without blood in the stool**
Either (A) or (B), or both
**(A)** - Acute watery diarrhoea (at least 3 loose stools in a day)
OR
- Vomiting

**(B)** Clinical suspicion of acute gastroenteritis, without blood in the stool.

**[3] Bloody diarrhoea**
Either (A) or (B), or both
**(A)** - Acute diarrhoea (at least 3 loose stools in a day)
AND
- Visible blood in the stool

**(B)** Clinical suspicion of acute dysentery.

**[4] Rash WITH fever**
Either (A) or (B), or both
**(A)** - Fever (≥38.0°C directly measured or reported by the patient)
AND at least one of the following:
- Rash (erythematous, macular, papular, vesicular, pustular rash or a combination of these)
- Enanthem (macular and/or vesicular)

**(B)** Clinical suspicion of measles, rubella, chickenpox (varicella), scarlet fever or other rash illness.

***Note:*** *Haemorrhagic rash (petechiae, purpura, ecchymoses) or enanthem with fever is recorded as syndrome [12].*

**[5] Suspected scabies**
Either (A) or (B), or both
**(A)** - Skin lesions caused by pruritus/scratching
AND
- Papules, vesicles or small linear burrow tracks

**(B)** Clinical suspicion of scabies.

***Note:*** *Head lice or other parasitic skin infections are not included.*

**[6] Suspected pulmonary tuberculosis**
Clinical suspicion of pulmonary tuberculosis (TB):
- Productive cough lasting more than 3 weeks

***Note:*** *Other symptoms consistent with TB: low-grade evening fever and/or night sweats lasting more than 3 weeks, asthenia, weight loss in the last 3 months.*

**[7] Malaria (with POSITIVE rapid test)**
Clinical suspicion of malaria and rapid diagnostic test performed which had a positive result.

***Note:*** *Clinical suspicion of malaria (rapid diagnostic test recommended): fever and/or malaise (with or without other symptoms, see below)* ***with*** *exclusion of other obvious causes* ***in persons*** *originating in an endemic country or with history of recent travel/residence in an endemic country or an area with local transmission of malaria.*
*Other symptoms or findings consistent with malaria: headache, chills, sweats, arthralgia/myalgia, nausea, vomiting, diarrhoea, cough, anaemia, thrombocytopenia, splenomegaly.*

**[8] Diphtheria, respiratory or cutaneous**
Clinical suspicion of respiratory or cutaneous diphtheria:
- Pharyngitis, tonsillitis or laryngitis with adherent membrane of the tonsils, pharynx and/or nares
- Skin ulcers

**[9] Jaundice of acute onset**
Either (A) or (B), or both
**(A)** Jaundice or sub-jaundice of acute onset
**(B)** Clinical suspicion of viral hepatitis, leptospirosis or yellow fever.

***Note:*** *Alcoholic liver disease is excluded.*

**[10] Paralytic manifestations of acute onset**
Either (A) or (B), or both
**(A)** - Acute onset of flaccid paralysis
OR
- Acute onset of cranial nerve paralysis: e.g. blurred vision, diplopia, ptosis, extraocular muscle weakness or paresis, dysarthria, dysphagia

**(B)** Clinical suspicion of poliomyelitis or botulism.

***Notes:*** *i) Acute paresis or paralysis due to trauma is not included. ii) If syndromes [10] and [11] coexist, the case is recorded as syndrome [10].*

**[11] Meningitis and/or encephalitis**
Either (A) or (B), or both
**(A)** - Fever (≥38.0°C directly measured or reported by the patient)
AND at least one of the following:
- Persistent headache
- Neck stiffness
- Altered mental status (e.g. disorientation, confusion, delirium)

**(B)** Clinical suspicion of meningitis or encephalitis, regardless of possible aetiology (e.g. viruses, bacteria, fungi or other factor).

**[12] Haemorrhagic manifestations WITH fever**
Either (A) or (B), or both
**(A)** - Fever (≥38.0°C directly measured or reported by the patient)
AND at least one of the following:
- Haemorrhagic rash (petechiae, purpura, ecchymoses)
- Haemorrhagic enanthem
- Bleeding from mucous membranes (gingival, conjunctival, epistaxis)
- Unexplained bleeding from any system

**(B)** Clinical suspicion of viral haemorrhagic illness.

***Notes****: i) Bleeding due to blood disorders or trauma is not included. ii) If syndromes [11] and [12] coexist, the case is recorded as syndrome [12].*

**[13] Sepsis or shock (septic, of unknown aetiology)**
Either (A) or (B), or both
**(A)** - Clinical signs or symptoms of infection from any organ/system
AND
- Clinical signs or symptoms of organ dysfunction/failure (e.g. severe hypotension)

**(B)** Clinical suspicion of sepsis or shock.

***Note****: Circulatory collapse due to congestive heart failure, acute myocardial infarction or trauma are not included.*

**[14] Death of unknown aetiology**
Death of unknown cause.

## Information in table (C)

| | |
|---|---|
| Column "#" | Sequential number of patient in the Reporting Form is included (e.g. 1, 2, 3, 4). If space for additional patients is needed, use another Reporting Form (in which only table (C) & initial info are filled in), and continue the sequential numbers (e.g. 5, 6, etc.). |
| Column "Patient Surname-Name, Dwelling, Contact telephone no." | This information is included in the printed version of the Reporting Form, so that the patient can be followed up and the necessary public health measures in his/her surrounding can be taken. **Only the patient's initials will be electronically transmitted.** The printed Reporting Forms will be safely kept with the responsibility of the Coordinator of the site. |
| Column "# of syndrome" | The corresponding number of syndrome or condition is filled in: [1] to [14]. |
| Column "Examining physician in site & Contact tel." | Physician's name and contact telephone are included, so that –if need arises– communication with him/her for clarifications on the condition of the patient is possible. |

*FORM UPDATE: 16 May 2016*
