# Supplementary material for: Clinical assessment is a neglected component of outbreak preparedness: evidence from refugee camps in Greece
Source: BMC Med. 2018 Mar 19;16:43. doi: 10.1186/s12916-018-1015-9 (PMC5858141; doi:10.1186/s12916-018-1015-9)
Supplement: Supplementary file 2 — Demographic data for included patients. (PDF 12 kb) [file 12916_2018_1015_MOESM2_ESM.pdf]

Rojek et al. Appendix 2.

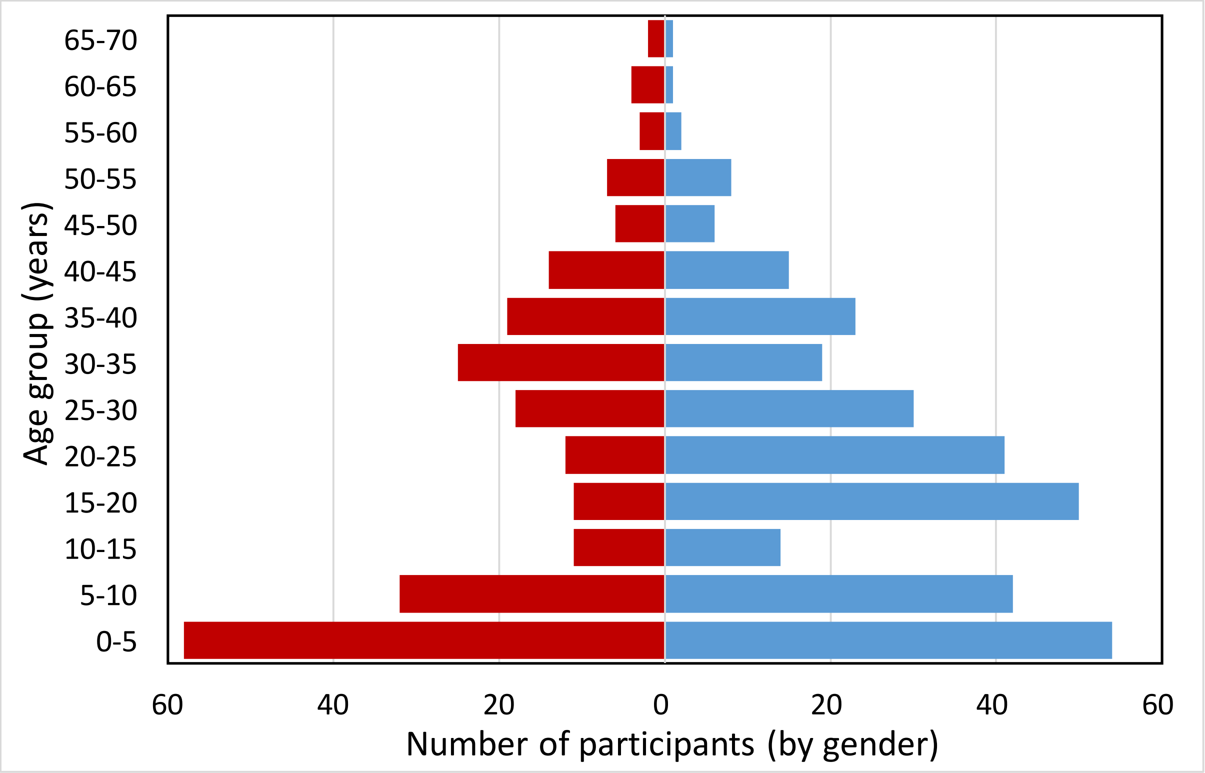

Figure s2: Age and gender of included participants. Blue describes male patients, and red describes female patients.
